# The Taming of Nuclear Factor Erythroid-2-Related Factor-2 (Nrf2) Deglycation by Fructosamine-3-Kinase (FN3K)-Inhibitors-A Novel Strategy to Combat Cancers

**DOI:** 10.3390/cancers13020281

**Published:** 2021-01-14

**Authors:** Narasimha M. Beeraka, Venugopal R. Bovilla, Shalini H. Doreswamy, Sujatha Puttalingaiah, Asha Srinivasan, SubbaRao V. Madhunapantula

**Affiliations:** 1 Center of Excellence in Molecular Biology and Regenerative Medicine (CEMR), Department of Biochemistry, JSS Medical College, JSS Academy of Higher Education & Research (JSS AHER), Mysuru, Karnataka 570015, India; bnmurthy24@gmail.com (N.M.B.); venu.1726@gmail.com (V.R.B.); shalini.hd98@gmail.com (S.H.D.); sujatha.p05@gmail.com (S.P.); 2 Public Health Research Institute of India (PHRII), Mysuru, Karnataka 570020, India; 3 Division of Nanoscience and Technology, Faculty of Life Sciences, JSS Academy of Higher Education & Research (JSS AHER), Mysuru, Karnataka 570015, India; asha.srinivasan@jssuni.edu.in; 4 Special Interest Group in Cancer Biology and Cancer Stem Cells, JSS Medical College, JSS Academy of Higher Education & Research (JSS AHER), Mysuru, Karnataka 570015, India

**Keywords:** glycation, deglycation, FN3K, Nrf2, RAGE, AGEs

## Abstract

**Simple Summary:**

Aim of this review is to provide an overview on (a) Fructosamine-3-Kinase (FN3K) and its role in regulating Nuclear Factor Erythorid-2-Related Factor-2 (Nrf2); (b) the role of glycation and deglycation mechanisms in modulating the functional properties of proteins, in particular, the Nrf2; (c) the dual role of Nrf2 in the prevention and treatment of cancers. Since controlling the glycation of Nrf2 is one of the key mechanisms determining the fate of a cell; whether to get transformed into a cancerous one or to stay as a normal one, it is important to regulate Nrf2 and deglycating FN3K using pharmacological agents. Inhibitors of FN3K are being explored currently to modulate Nrf2 activity thereby control the cancers.

**Abstract:**

Glycated stress is mediated by the advanced glycation end products (AGE) and the binding of AGEs to the receptors for advanced glycation end products (RAGEs) in cancer cells. RAGEs are involved in mediating tumorigenesis of multiple cancers through the modulation of several downstream signaling cascades. Glycated stress modulates various signaling pathways that include p38 mitogen-activated protein kinase (p38 MAPK), nuclear factor kappa–B (NF-κB), tumor necrosis factor (TNF)-α, etc., which further foster the uncontrolled proliferation, growth, metastasis, angiogenesis, drug resistance, and evasion of apoptosis in several cancers. In this review, a balanced overview on the role of glycation and deglycation in modulating several signaling cascades that are involved in the progression of cancers was discussed. Further, we have highlighted the functional role of deglycating enzyme fructosamine-3-kinase (FN3K) on Nrf2-driven cancers. The activity of FN3K is attributed to its ability to deglycate Nrf2, a master regulator of oxidative stress in cells. FN3K is a unique protein that mediates deglycation by phosphorylating basic amino acids lysine and arginine in various proteins such as Nrf2. Deglycated Nrf2 is stable and binds to small musculoaponeurotic fibrosarcoma (sMAF) proteins, thereby activating cellular antioxidant mechanisms to protect cells from oxidative stress. This cellular protection offered by Nrf2 activation, in one way, prevents the transformation of a normal cell into a cancer cell; however, in the other way, it helps a cancer cell not only to survive under hypoxic conditions but also, to stay protected from various chemo- and radio-therapeutic treatments. Therefore, the activation of Nrf2 is similar to a double-edged sword and, if not controlled properly, can lead to the development of many solid tumors. Hence, there is a need to develop novel small molecule modulators/phytochemicals that can regulate FN3K activity, thereby maintaining Nrf2 in a controlled activation state.

## 1. Introduction

**Figure 1:**
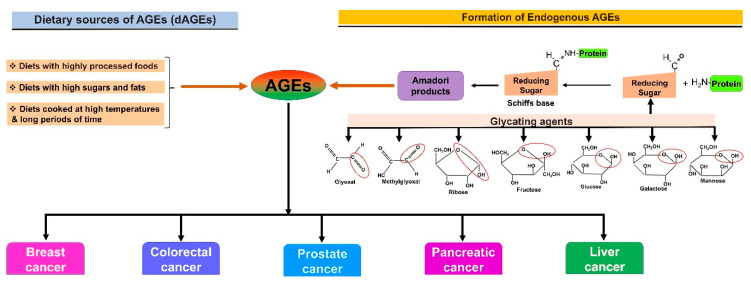
The mechanism of the formation of advanced glycation end products (AGEs): Glycation of proteins is mediated by the reaction between amino (-NH_2_) groups of amino acids, especially lysine residues, and the carbonyl group of sugars (-CR=O or –CH=O), leading to the generation of products through the Maillard reaction. The generated Maillard reaction products subsequently undergo Amadori rearrangement to form advanced glycation end products (AGEs) that are implicated in cancer progression.

Glycation and Cancers: Glycation is a nonenzymatic addition of carbohydrates such as glucose and fructose to the target proteins and lipids by the covalent bond formation [1,2]. Glycation is one of the key cellular mechanisms involved in controlling the progression and drug resistance in cancer cells [1,3]. The formation of advanced glycation end products (AGEs) occurs through the glycation of proteins and lipids (Figure 1) [3]. In particular, reducing sugars (ex., glucose and fructose) react with the amino groups of macromolecules, especially the proteins and lipids, which consequently facilitate the formation of AGEs; this reaction is referred to as a Milliard reaction and involves the formation of Amadori products [4,5,6]. However, the interplay between the receptors for AGEs—in short, known as RAGEs—and AGEs modulate several cell signaling pathways in mediating cancer progression [3]. Studies have demonstrated that AGEs can promote oxidative stress, thereby increasing the expression of transcription factors, proinflammatory and inflammatory cytokines, and acute phase proteins [7]. Furthermore, the accumulation of AGEs and their binding to RAGEs can cause metabolic disorders, inflammation, and oxidative stress [7].

**Figure 2:**
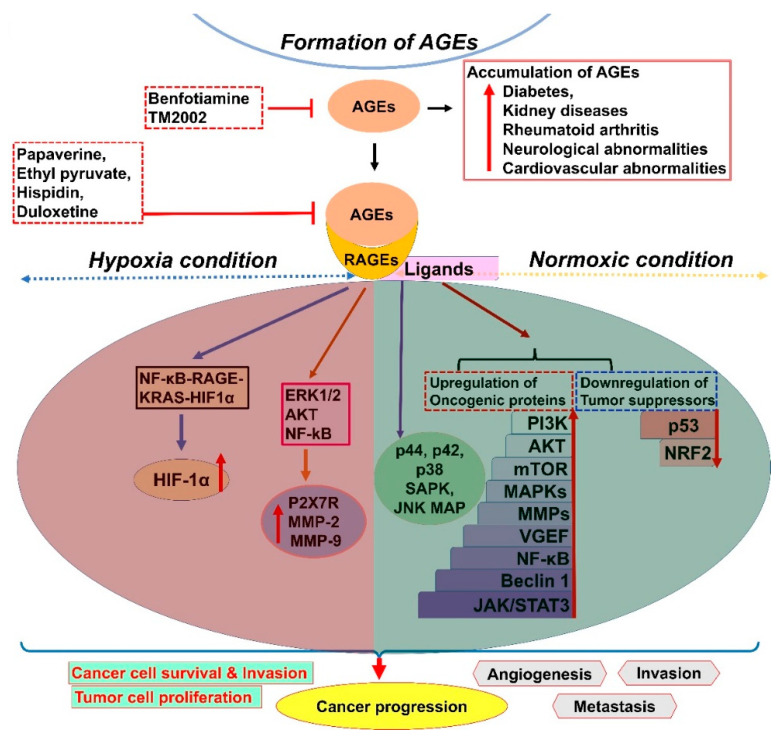
Molecular mechanisms involved in mediating tumor progression through AGEs and receptor for AGE (RAGE)-mediated signaling cascades. Glycation of Nrf2, NF-κB, p53, Akt, mTOR, MAPKs, MMPs, VEGF, Beclin 1, and JAK/STAT3 modulates the activity of these proteins, thereby controlling the cancer progression. RAGE inhibitors, viz., duloxetine, papavarine, hispidin, and ethyl pyruvate, could efficiently block the activity of these proteins. Furthermore, hypoxia-induced AGE-RAGE signaling is conducive to the modulation of Akt, ERK1/2, and NF-kB followed by the changes in the expression of MMP-2 and MMP-9 to foster cancer cell survival and invasion. Deregulated AGE signaling is also conducive to the risk of developing diabetes, renal diseases, rheumatoid arthritis, and cardiovascular and neurological abnormalities. AP-1: activator protein 1, p53: tumor protein p53/phosphoprotein p53/tumor suppressor p53, HIF-1α: hypoxia-inducible factor 1-alpha, STAT3: signal transducer and activator of transcription 3, PPAR-γ: peroxisome proliferator-activated receptor gamma, PI3K: phosphoinositide 3-kinase, IL-1β: interleukin-1β, IL-6: interleukin 6, TNF-α: tumor necrosis factor-alpha, VCAM-1: vascular cell adhesion molecule 1, and ICAM-1: intercellular adhesion molecule 1.

RAGEs belong to the immunoglobulin superfamily of cell surface proteins, and AGE–RAGE interactions can foster the alteration of several downstream signaling pathways [8,9,10]. Glycation and RAGEs are involved in the pathogenesis and progression of several cancers by enhancing metastasis, invasion, and angiogenesis (Figure 2 and Table 1) [2,11,12]. Recent studies have delineated the interaction of RAGEs with a wide range of acidic ligands, viz., AGEs, S100s, high-mobility group box1 (HMGB1), and their role in promoting cancer. For instance, the RAGE–ligand interactions could effectively induce antiapoptotic and proapoptotic protein expression through the upregulation of PI3K/protein kinase B (Akt)/mammalian target of rapamycin (mTOR), mitogen-activated protein kinases (MAPKs), matrix metalloproteinases (MMPs), vascular endothelial growth factor (VEGF), and nuclear factor kappa B (NF-κB) pathways. However, these ligand interactions induce the downregulation of p53 expression, consequently inflicting cancer progression [13]. Furthermore, RAGE–ligand interactions can form multimodal dimerization, which eventually induces cancer progression; hence, the inhibition of RAGEs could effectively control cancer progression [13]. Clinical studies have delineated that determining the RAGE levels in body fluids could be used as novel biomarkers for several cancers, viz., lung cancer [14], breast cancer [15], prostate cancer [16], and colorectal cancer [17,18]. Recent studies have demonstrated that the targeted inhibition of RAGE-signaling cascades using papaverine, ethyl pyruvate, hispidin, duloxetine, etc. could retard gliomas and sarcomas [13,19,20].

The dietary consumption of AGEs is likely to enhance the risk of pancreatic cancer in males. However, the females consuming a diet rich in CML AGEs (Nϵ-(carboxymethyl) lysine) have not acquired the risk of pancreatic cancer [27].

Hypoxia condition could also confer the accumulation of AGEs in cancer cells and mediate the progression of cancers [28]. AGE-RAGE interaction actuates several signaling mechanisms to promote cancer at the time of oxygen deprivation conditions in tumor microenvironment. For example, hypoxia-induced RAGE upregulation was significantly reported in the hepatocellular cancer cells [29]. A study by Welford SM net al (2011) reported enhanced phosphorylation of Erk1/2 and Akt; and translocation of NF-kB into the nucleus by hypoxia-mediated RAGE, which consequently enhanced cancer cell survival and invasion through the modulation of ATP-gated ionotropic P2X7 receptor (P2X7R) and MMP-2 and MMP-9 expression [30]. RAGE-mediated HIF-1α activation occurs during hypoxia through the modulation of NF-κB—RAGE—Kirsten Rat Sarcoma viral oncogene homologue (KRAS)—HIF1α signaling in pancreatic cancer cells [31]. Therefore, the pharmacological interventions targeting AGE-RAGE for treating hypoxic cancer cells are gaining more attention by oncologists. For example, Benfotiamine-mediated AGE blockade was reported as an effective therapeutic approach in type 2 diabetes; however, the efficacy of this drug for treating hypoxia-induced cancers is yet to be examined in clinical settings [3,32]. TM2002 is another potent inhibitor of AGE reported to be protective against renal injury and cardiovascular diseases. However, the efficacy of TM2002 is yet to be examined against AGEs in hypoxia-driven cancers [33]. Furthermore, several pharmacological interventions have been developed to target these AGE-RAGE interactions, as well as the signaling mechanisms, in cancers to design effective therapeutic modalities [3].

Prolonged hyperglycemia is the major contributing factor for the occurrence and pathophysiology of glycation events [34,35]. The end products of glycation, viz., glyoxal, methylglyoxal (MGO), and 3-deoxyglucosone (3-DG), are toxic intermediates that are known to contribute to the development of hepatocellular carcinoma (HCC) [36,37,38]. In addition, the AGEs also confer oxidative stress by producing reactive oxygen species (ROS) and reactive nitrogen species (RNS), which, in turn, impair redox homeostasis and modify the structures of various proteins that include insulin, Nrf2, oligosaccharyl transferase-48 (OST-48), and galectin-3 [7]. Furthermore, glycation events often lead to the impairment of superoxide dismutase (SOD) activity, hence causing oxidative damage through the production of peroxide radicals [39].

AGE–RAGE mediated signaling stimulates reduced nicotinamide adenine dinucleotide phosphate (NAD(P)H)-oxidase via the protein kinase-C and mitochondrial electron transport system [40,41]. AGEs-mediated oxidative stress is orchestrated through NF-kB-dependent signaling by activating TNF-α expression, which binds to tumor necrosis factor receptor-1 (TNFR-1) [42,43,44]. Glycative stress and carbonyl stress are involved in a spectrum of modulatory activities in several cell signaling pathways to enhance cancer progression, tumor growth, and multidrug resistance [45]. Dicarbonyl glycation invokes a significant influence on tissue invasion and metastasis due to the elevated flux of MGO and Glo1 (Glyoxalase 1) expression and other reactive α-oxoaldehyde metabolites [45]. These metabolites could confer a wider spectrum of metabolic reprogramming (Warburg effect) in tumor cells [46,47,48]. Transcriptome-wide gene expression analysis delineated the influence of extensive Glo1 expression on cancer cell lines sensitive and resistance to chemotherapeutic drugs [49,50]. A report by Naila Rabbani et al. (2017) described the cause of multidrug resistance in tumor cells through the Nrf2 signaling due to the high glycolytic activity and Glo1 expression [45].

Redox signaling is mediated by the toxic free radicals, viz., peroxides, hydroxyl (•OH), and superoxide (O2•–) generated from endogenous metabolic activities [51,52]. An extensive amount of ROS is generated inside the cell from the mitochondrial respiratory chain. Further, hypoxia condition also triggers the mitochondrial respiratory chains to generate RNS, which could enhance the production of malondialdehyde (MDA) and 4-hydroxynonenal (4-HNE) through lipid peroxidation [52,53]. AGE–RAGE signaling could invoke a sustained activation of ROS generation in cancer cells [54]. At moderate concentration, ROS can actuate several cancer cell survival signaling cascades, viz., MAPK/ERK1/2, P38, JNK (c-Jun N-terminal kinase), and PI3K/Akt, which, in turn, trigger the activation of NF-kB, VEGF, and MMP activation [55]. However, at high concentrations, ROS could invoke cell death in cancer cells through mitochondria-mediated apoptosis via either the intrinsic or extrinsic pathway [55]. Furthermore, glycation-mediated oxidative stress facilitates the activation of several transcription factors, viz., nuclear Nrf2, NF-κB, p-53, AP-1, HIF-1α, STAT3, β-catenin/Wnt, and PPAR-γ [56] (Figure 2). These activated factors can trigger gene expression to modify several inflammatory cytokines and growth factors and simultaneously induce cancerous outgrowth [56,57]. Carbonyl stress can induce damage to the cellular constituents, viz., proteins, sugar molecules, DNA, and lipoproteins, consequently promoting cancer growth [56]. A report by Lin JA et al. (2016) delineated that the AGEs levels in body fluids could be used as independent determinants of inflammatory markers as the levels of circulating AGEs positively correlated to inflammatory markers (ex., C-reactive protein), endothelial dysfunction, and vascular complications [58]. Several other scientific reports described that there is a correlation between cigarette smoking and AGEs-induced glycated stress; the exogenous AGEs generated due to heavy smoking could be conducive to the divergent oncogenic signaling across the tissues and foster the risk of acquiring carcinomas of colon and rectum, liver, and pancreas [59,60]. Glycated and carbamylated type I collagen facilitate the uncontrolled proliferation of invasive human HT1080 fibrosarcoma cells [61]. Glycated collagen I mitigates the cell adhesion time and confers tumor metastasis [61]. In addition, AGEs can induce modifications in the basement membrane to facilitate the metastasis of prostate and breast cancers [10,56,62]. RAGE–NADP+ oxidase (NOXs) interactions foster oxidative damage, tumor hypoxia, and the activation of VEGFs to further promote tumor angiogenesis [63].

RAGE polymorphism is another significant factor that contributes to the incidence of several cancers, viz., oral squamous cell carcinoma (OSCC) and lung and breast cancers [64,65,66,67]. Single nucleotide polymorphism (SNPs) rs1800625 of the RAGE genes is reported to be involved in the development of OSCC [65]. The interactions between environmental mutagens and RAGE gene polymorphisms are significant predisposing factors to foster OSCC [65]. A report by Hongmei Wang et al. (2015) delineated a significant association between the RAGE SNPs-429T/C and 2184A/G to promote lung cancer [68]. Another report by Tesarova et al. (2017) unraveled the association between breast cancer with G82S and 2184 A/G RAGE SNPs [69]. Furthermore, AGEs–RAGEs are reported to be involved in enhancing the expression of cell survival proteins, viz., p38 mitogen-activated protein kinase (MAPK), to facilitate cancer cell proliferation and survival [23,70].

Thus, glycation exhibits significant implications in promoting cancers, and, on the contrary, deglycation of certain proteins such as Nrf2, particularly in preventing cancers, may deliver effective clinical outcomes, which must be tested in clinical settings. In this review, we provided a balanced view of glycation in cancer progression by modulating several cell signaling pathways and discussed the role of deglycation by FN3K and the need for the development of FN3K inhibitors to maintain Nrf2 in glycated state in order to effectively treat advanced cancers.

## 2. AGE–RAGE Signaling and Cancer Progression

AGEs can enhance the migration of cancer cells by promoting the activity of VEGF, NF-κB, and ERK signaling pathways [13,24,25]. In cancers such as colorectal cancer (CRC), breast, oral, and prostate cancers, AGE–RAGE signaling could promote cancer cell proliferation. For instance, the S100 proteins are particularly reported to be associated with the RAGE signaling pathway to mediate cancers, viz., melanoma, osteosarcoma, CRC, and breast carcinomas [13].

Furthermore, AGE–RAGE signaling is not only involved in enhancing ROS induction but also triggers the generation of several inflammatory factors, viz., Nrf2, NF-kB, HIF1a, and STAT3 [71]. These inflammatory factors further can modulate the secretion of cytokines such as IL-1β, IL-6, and TNF-α and foster the production of cell adhesion molecules, viz., VCAM-1, ICAM-1, and endothelin-1 and mitigates the production of endothelial nitric oxide synthase (eNOS). These factors actively modulate the immune/myeloid cell recruitment (ex., tumor-associated macrophages (TAMs)) to promote angiogenesis towards growing tumor cells [71,72] (Figure 2). AGE–RAGE signaling is also involved in regulating the activity of carbohydrate response element binding protein (ChREBP), a metabolic transcription factor in the liver and colon cancer cells. Thus, the proliferation rate of these cells is also modulated by glycation end products [73,74]. RAGE-mediated tumor responses are triggered by the STAT3 and NF-kB transcription factors, which, in turn, enhance tumor metastasis [75,76] (Table 1). The glycation events associated with AGE-RAGE signaling predominantly confer the tumor cell proliferation in several cancers, viz., oral, HCC, renal, breast, and leukemia and skin cancers [45]. In addition, this signaling also mediates the invasion, metastasis, and angiogenesis of all these cancers [67]. AGEs, in association with RAGEs, could also induce the translocation of High Mobility Group Box-1 (HMGB1) to foster the formation of aggressive and invasive tumor phenotypes in colon adenomas [56,77,78]. HMGB1 is a transcription factor, which, along with amphoterin, effectively binds to RAGEs to promote the cancer cell proliferation, invasion, and VEGF production in colon cancer cells [25,77]. Extracellular HMGB1 can also bind to TLR2 (Toll-like receptor-2), TLR4, and RAGEs, and the impediment of RAGE–HMGB1 interaction could block the activation of p44/p42, p38, and Stress-activated protein kinases (SAPK)/JNK MAP kinases, which were significantly conducive to tumor proliferation [78,79,80]. Hence, prospective research should focus on the development of therapeutic molecules, novel small molecule inhibitors (NSMIs), to target these signaling pathways pertaining to the glycation-mediated induction of Nrf2, NF-kB, HIF1a, and STAT3 using in vitro and in vivo models.

## 3. Role of Glycation in the Modulation of Target Protein Expression and Activity

Deglycation pathways are reported to be the significant counteracting mechanisms against stochastic free radical-mediated oxidative damage [81]. The natural antioxidant defense mechanisms can control ROS damage by mitigating the production of ROS and RNS [81]. For instance, ascorbic acid; tocopherol; and antioxidant enzymes, viz., peroxidase, catalase, superoxide dismutase (SOD), and glutathione peroxidase (GPx), are strong antioxidant defenses to mitigate AGE-mediated oxidative stress [82,83,84]. It is imperative to investigate counteracting mechanisms through the development of NSMIs to limit glycation through the upregulation of deglycation mechanisms. For instance, fructosamine formation is a stable and irreversible mechanism, which further leads to the production of AGEs to promote cancer growth [85,86]. On the contrary, a broad scientific research focused on the glycation process has generated an array of specific reports since the breakthrough discovery and cloning of a putative deglycating enzyme, FN3K [81,85,86]. FN3K-mediated deglycation of Nrf2 could confer cancer progression in HCC [87]. Hence, future research should focus on the development of NSMIs to modulate these glycation/deglycation processes to mitigate cancer progression.

Deglycation has a physiological significance, as this process can avoid the formation of AGEs from fructosamines [87]. FN3K can act on the glycated moieties that are formed from the covalent crosslinking of proteins, thereby promoting deglycation. Another functional aspect of deglycation is to prevent the loss of amino acid residues required for protein/protein interactions [87,88,89]. The significant difference between DNA repair system and protein repair system is the accessibility for these residues. For example, modified bases are equivalently accessible in the case of DNA repair [90]; however, only a fraction of amino acid residues is accessible in the case of a protein repair system through deglycation. The “fructolysine residues” of a protein are expected to be accessible and undergo deglycation by FN3K with similar efficiencies in a FN3K hypothetical protein unfolding system [87,88,89]. However, further studies are warranted to confirm this function in deglycation.

## 4. Nrf2 and Its Glycation and Deglycation Mechanisms in Regulation of Cancers

### 4.1. Nuclear Factor Erythroid-2-Related Factor 2 (Nrf2)

**Figure 3:**
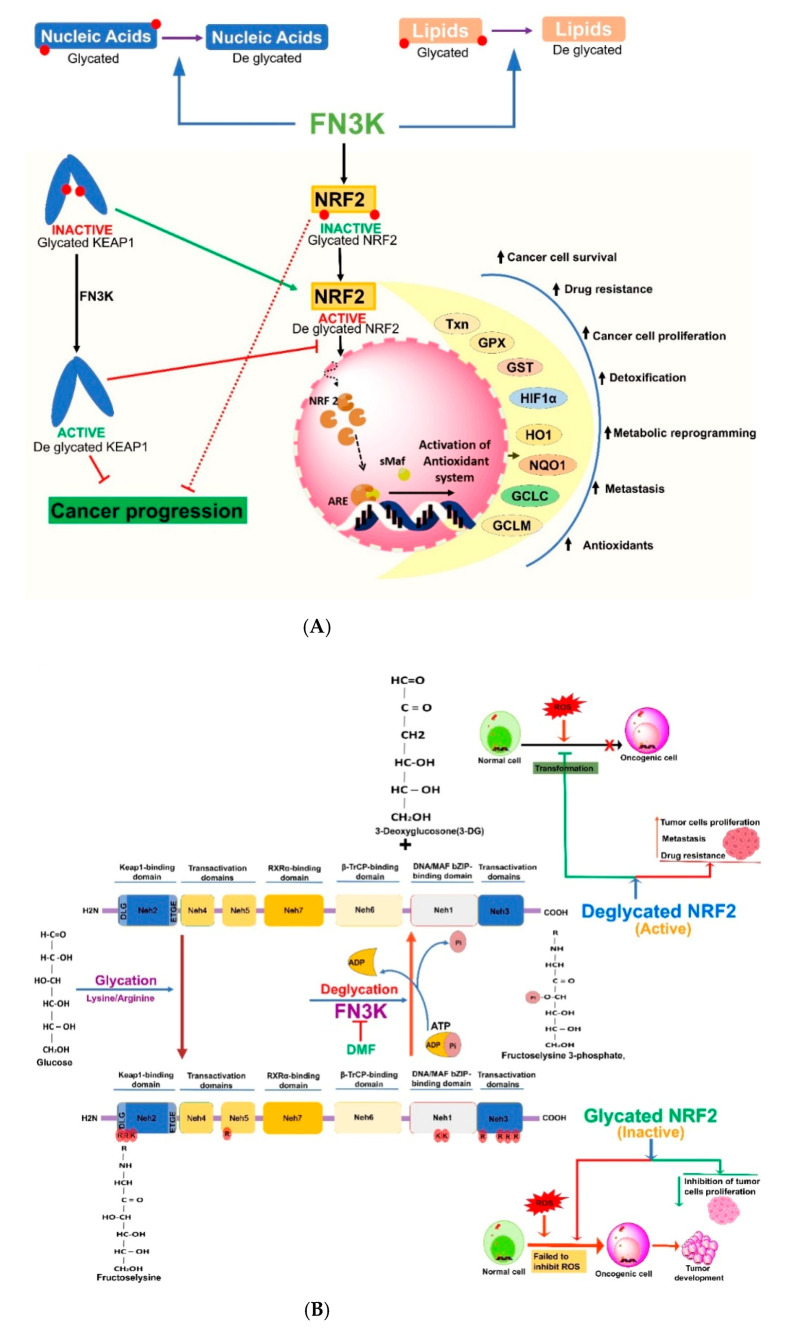
(**A**) Signaling pathways involved in the regulation of Nrf2: Oncogenic Nrf2 signaling is regulated by glycation and deglycation mechanisms in cancer cells. The redox regulator Nrf2 is an oncogenic transcription factor involved in controlling cellular oxidative stress, cell proliferation, migration, drug resistance, and metastasis. Nrf2 and its negative regulator Keap1 are controlled by glycation and deglycation mechanisms. (**B**) Schematic representation of glycated Nrf2: Glycated Nrf2 containing various binding domains from Neh1 to Neh7 where glycation occurs (indicated in small red circles) is depicted in this schematic representation. FN3K is a deglycating enzyme reported to promote functionally activate the Nrf2 level in cancer cells and enhance cancer progression. Fructolysine residues of glycated Nrf2 react with ATP and get converted into a phosphorylated form in the presence of FN3K. The blockade of FN3K could promote the formation of glycated Nrf2 and mitigation in oncogenicity and cancer progression. Txn—thioredoxin, GST—glutathione S-transferase, HO1—heme oxygenase-1, NQO1—NAD(P)H:quinone oxidoreductase 1, GCLC—glutamate-cysteine ligase catalytic subunit, GCLM—glutamate cysteine ligase modulatory gene, sMaf—small Maf proteins, and ARE—antioxidant response element.

Nrf2 is a leucine zipper transcription factor involved in the activation of antioxidant response genes (ARE) in stress conditions by binding to the cis-regulatory genes inside the nucleus of a cell (Figure 3) [91,92]. Nrf2 is composed of seven highly conserved domains from Neh1 to Neh7; among them, Neh1 specifically contains a bZIP domain for DNA binding and sMAF for protein binding. The Neh2 domain is required to mediate the interaction between Keap1 (KELCH-like ECH-associated protein 1) with specific Neh2 amino acid binding sites, viz., DLG (^22^ILWRQDIDLGVSREV^36^) and ETGE (AFFAQLQLDEETGEFL)motifs [92]. In normal conditions, DLG and ETGE motifs of Nrf2 bind to Keap1 and undergo polyubiquitylation and proteasomal degradation in the cytosol [92]. Thus, these motifs are crucial to mediate KeaP1-dependent Nrf2 proteasomal degradation through the ubiquitylation of lysine residues by the Cul3/Rbx1/E3 complex. Specifically, Neh2 acts as a negative regulator of Keap1, and the regulation of Nrf2 levels by Keap1 is abrogated in human cancers [92]. The Neh3-5 domains are essential for target gene transactivation in the presence of several modulators. The Neh6 domain is composed of serine-rich amino acid regions to foster Nrf2 degradation [92]. Keap1 is another significant component, which contains five domains, viz., the (i) N-terminal domain (NTD), (ii) bric-ábrac (BTB) homodimerization domain involved in fostering the interaction of Neh2 with Nrf2, (iii) cysteine-rich intervening (IVR) domain, (iv) a double-glycine repeat (DGR) domain with six Kelch motifs, and (v) a C-terminal region (CTR) [93,94].

ROS-mediated oxidative stress could induce damage to polyunsaturated fatty acids, followed by the release of diffusible electrophilic α,β-unsaturated aldehydes, such as 4-hydroxynonenal [95]. During these electrophilic stress conditions, Nrf2 turnover is maintained through Keap1-mediated proteasomal degradation via two-site substrate recognition mechanisms. Consequently, the two Nrf2-Keap1-binding sites together form a hinge and latch structure. The ETGE and DLG motifs of Nrf2 are recognized by the E3 ligase adaptor Keap1 [95]. Thus, the electrophile oxidants could cause redox modification in the reactive cysteine residues, followed by the induction of conformational changes in Keap1, which subsequently impedes Nrf2 ubiquitination [96,97]. Thus, the nascent Nrf2 escapes from the Keap1-dependent degradation and undergoes translocation into nucleus to form heterodimers with sMAFs and binds to AREs or electrophilic-responsive elements of the promoter region of DNA to foster the transactivation of cytoprotective genes, viz., NAD(P)H:quinone oxidoreductase 1 (NQO1) of Phase I and heme oxygenase (HO)-1) of Phase II, glutathione S-transferase (GST), UDP-glucuronosyltransferase (UGT), and UDP-glucuronic acid (UGA) of Phase II and phase III enzymes [92,98,99,100]. Thus, during oxidative/electrophilic stress conditions, Nrf2/Keap1 launches several cytoprotective effects through the binding to the small MAF proteins [92]. In addition, Nrf2 activation induces the transcription of cytoprotective genes for phase II xenobiotic detoxification, drug efflux, glutathione homeostasis, and proteome maintenance [91,101]. Inactivating mutations in Keap1 have been reported to induce several cancers, including breast cancer and non-small cell lung carcinoma (NSCLC), as well as squamous cell lung carcinomas (SCLCs), further actuating the tumorigenicity and resistance to chemotherapy [102,103]. In addition, Nrf2 has its own implications in tumor biology, as it enhances drug efflux proteins, apoptosis suppressors, and redox balance modulators, consequently protecting cancer cells from drug therapy, radiotherapy, and apoptotic inducers [91,92] (Figure 3A).

Nrf2 is encoded by nuclear factor erythroid-derived 2-like 2 gene (NFE2L2), which can further regulate the antioxidant and redox stress (carbonyl, glycated, and deglycated), leading to cancer and chemotherapeutic drug resistance [104,105,106,107,108,109,110,111]. Normally, Nrf2 confers the transcription of ARE-bearing genes responsible for glutathione (GSH) synthesis, redox homeostasis, the detoxification of xenobiotics, and anabolic metabolism [110]. Further, a plethora of reports delineated that the key regulator for Nrf2 activity is Keap1. Mutated Keap1 was reported in several cancers, viz., lung cancer, HCC, endometrial cancer, bladder cancer, colon cancer, head and neck cancer, and esophagogastric cancer [110,112]. GSH generation can effectively neutralize the ROS produced at the time of oncogene-mediated cancer cell proliferation when exposed to alkylating drugs or radiation [113,114,115,116]. Consequently, extensive ROS neutralization can prevent breast cancer and colon cancer development [117]. While the loss of Keap1 fosters KRAS (a proto-oncogene GTPase)-mediated lung cancer, previous studies proved Nrf2-driven protection in vivo against carcinogen-induced lung cancer [109,118,119,120,121]. According to The Cancer Genome Atlas (TCGA) reports, the exclusive mutations were reported in the Nrf2, E3 ubiquitin ligase complex, Keap1, cullin3 (CUL3), and Cullin-associated NEDD8-dissociated protein 1 (CAND1) factors in hepatocellular carcinoma (HCC) [122,123].

Nrf2 is a key regulator of metabolism in cancer cells: Cancer cells acquire a resistance to oxidative, metabolic, and therapeutic insults through Nrf2/Keap1 signaling, which results in cytoprotective responses [124]. Metabolic reprogramming in cancer cells is typically correlated to the regulation of redox homeostasis, indicating that blocking the Nrf2 mediated metabolic network may be beneficial to impairing the growth of solid and hematological cancers [124]. For instance, metabolic reprogramming in cancer cells facilitated by mitochondria-mediated redox balance is linked to Nrf2 activity. Nrf2 could influence the substrate availability during the electron transport chain of the mitochondrial metabolism; further, the process of mitochondrial dynamics and biogenesis fission/fusion are affected in cancer cells [125,126]. Nrf2 signaling is reported to be involved in fostering the alterations in the turnover and mitochondrial network dynamics involved in tumor adaptation to harsh conditions. For example, Nrf2 could alter the downstream IGF-1 (insulin-like growth factor 1) metabolic signaling involved in apoptosis and mitophagy through the modulation of BNIP3 (BCL2/adenovirus E1B 19-kDa protein-interacting protein 3) activity. This metabolic reprogramming was delineated in several cancer cells, viz., prostate, osteosarcoma, and breast cancer cells [127]. IGF-1 impaired the degradation of Nrf2 through the GSK-3β phosphorylation mediated via PI3K-Akt. Thus, the nuclear stagnation of Nrf2 occurs extensively to promote the BNIP3 induction that confers an alteration in mitochondrial turnover and biogenesis in cancer cells [127].

Nrf2 can also actively regulate cancer cell fatty acid metabolism. For instance, Nrf2 could regulate the fatty acid oxidation and mitochondrial respiration in HEK-293T cells by controlling the expression levels of mitochondrial carnitine palmitoyltransferase isoforms (CPT1 and CPT2) and other gene expressions, viz., acyl-CoA oxidase 1 and 2 (ACOX1 and ACOX2) [128,129]. Nrf2 also confers changes in the mRNA expression levels of hepatic enzymes, viz., Elovl2,3,5,6 and Cyb5r3 and ATP-citrate lyase fatty acid synthases, but their effects are not validated yet in cancer cell models [124].

Furthermore, Nrf2 has a significant role in the regulation of cancer cell amino acid metabolism pertaining to the transcription of genes involved in the coding of phosphoserine aminotransferase (PSAT1) and Serine Hydroxymethyltransferase 2 (SHMT2), the key enzymatic protein reported to be involved in serine/glycine biosynthesis via ATF4 activation in NSCLC cancer cell [130]. Nrf2 activity is essential for the modulation of ATF4 pathways through the KRAS pathway by inducing nutrient deprivation via PI3K/Akt signaling in NSCLC cells [131]. Hence, the therapeutic modalities to inhibit KRAS-Nrf2-ATF4 signaling in these cancer cells may be a beneficial strategy to establish in clinical models [124]. Nrf2-addicted cancer cells could foster the cysteine uptake, i.e., coordinated with glutamate excretion and glutathione generation, which, consequently, limits the glutamate involved in anaplerosis of the Krebs cycle and reduces mitochondrial respiration. Finally, cancer cells rely upon the exogenous nonessential amino acids for their metabolism. These metabolic aspects provide significant clues to develop novel therapeutic modalities against Nrf2-addicted cancer cells [124].

Nrf2 regulated redox homeostasis via the modulation of NADPH synthesis implicated in cancer cell antioxidant systems [124]. Mainly, Nrf2 activity directly or indirectly modulates the NADP+-dependent metabolic enzymes involved in the folate cycle or Krebs cycle or pentose phosphate pathway in Nrf2-addicted cancer cells. For instance, Nrf2 can confer the gene expression coding for NADPH-generating enzymes, viz., G6PD, PGD (phosphogluconate dehydrogenase), TKT (transketolase), and TALDO1 (transaldolase 1) in A549, H2126, and LK-2 lung cancer cells, and these genes are involved in cancer cell proliferation with a constitutive activation of PI3K/Akt signaling [132]. In summary, metabolic reprogramming mediated by Nrf2 plays an important role in the aggressive behavior of tumor cells, as well as their resistance to various chemotherapeutic agents. Therefore, strategies targeting these reprogrammed metabolic signaling cascades, along with Nrf2 inhibition, might yield a fruitful outcome. Hence, future research should focus on developing better therapeutic strategies aiming at co-targeting Nrf2 and deregulated metabolic pathways in cancer cells.

### 4.2. Nrf2 Role in Cancers: A Double-Edged Sword

The targeted activation of Nrf2 protects normal cells from various carcinogens: Nrf2 controls the antioxidant stress responses and drug detoxification by activating the synthesis of Phase I, II, and III drug detoxification enzymes, which eliminates the pro-oxidant molecules [133]. Thus, cellular homeostasis is being maintained during harmful internal and external cues. Phase I enzymes, viz., NQO1, carbonyl reductases, aldo-keto reductases, aldehyde dehydrogenase 1, and cytochrome P450 oxidoreductases cytochrome P450 proteins, are involved in mediating oxidoreduction and hydrolytic reactions of several xenobiotics [134]. Unlike Phase I enzymes, the Phase II enzymes HO-1, GST, UGT, and UGA catalyze the conjugation reactions [135]. The Phase III enzymes facilitate the transport of conjugated metabolites that are generated during Phase II reactions. Examples of Phase III proteins are multidrug resistance-associated proteins (MDR), breast cancer-resistant protein (BCRP), ATP-binding cassette g5 (ABCG5), and g8 (ABCG8) [134].

GSH synthesis is another significant pathway invoked by Nrf2 signaling, and the two main catalytic and modulator subunits of the glutamate–cysteine ligase and glutathione synthetase are major targets of Nrf2 to foster GSH production during cellular homeostasis [136]. Other Nrf2 targets involved in ROS-mediated oxidative stress are the redox cycling enzymes, viz., thioredoxin, glutathione peroxidase, superoxide dismutase 1, catalase, peroxiredoxin, sulfiredoxin etc., These cytoprotective proteins generated by Nrf2 protect a cell from various oxidative insults, including carcinogen-induced cancers [136]. Nrf2 thus inhibits tumorigenesis. For example, Nrf2 is reported to be activated by tumor-suppressor genes such as BRCA1 and p21 by blocking the formation of the Keap1/Nrf2 complex [108,137]. Further, Nrf2-deficient mice that are exposed to carcinogens developed tumors in the liver, lung, and bladder rather than wild-type mice, concluding that Nrf2 renders a protection against carcinogenesis. Several phytochemicals, such as curcumin, carnosol, resveratrol, sulforaphane, and synthetic chemicals like oltipraz, have proven their efficacy to activate Nrf2/ARE-regulated genes to mitigate tumorigenesis [138,139]. Activated Nrf2 could induce the generation of IL-17D expression and enhance NK cell recruitment to foster antitumor immunity [140]. In summary, the targeted activation of Nrf2 signaling is beneficial, as it prevents the transformation of a normal cell into a tumor cell. However, the administration of Nrf2 activators was proven to be pro-tumorigenic, hence requiring intensive research into the Nrf2 functions in specific immune cell subsets. Therefore, the modulation of Nrf2 through glycation could be an alternate approach to prevent this pro-tumorigenic role of Nrf2 [133].

**Figure 4:**
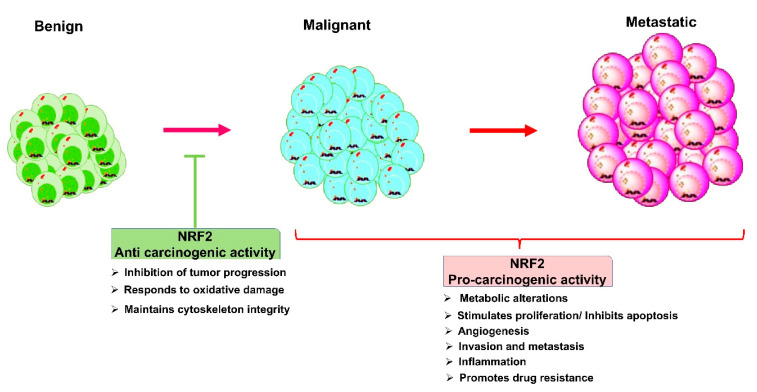
The dual role of Nrf2 in cancers: Nrf2 is a double-edged sword, which confers both anticarcinogenic activity and pro-carcinogenic functions. Nrf2 acts as a tumor-suppressive protein by blocking the transformation of benign cancer cells into malignant cancer cells and maintaining cytoskeleton integrity and impairing tumor progression. As an oncogenic protein, Nrf2 suppresses apoptotic cell death mechanisms, switches the metabolisms of cancer cells to anaerobic glycolysis and HMP shunts, and enhances angiogenesis. As a result, Nrf2 fosters cell proliferation invasion, metastasis, and drug resistance.

Functionally active Nrf2 protects even cancer cells, thereby promoting tumor growth and metastasis. While protecting normal cells, Nrf2 signaling also supports several transformed cells by enhancing the protection to cancer cells from oxidative damage, thereby facilitating their survival, migration, invasion, and progression to highly advanced stage tumors (Figure 4). Nrf2 activation promotes the migration and invasion of cancer cells through the activation of transcription factor BTB and CNC homology 1 (BACH1), consequently promoting tumorigenesis [141,142,143].

Nrf2 overexpression can induce the activation of glucose-6-phosphate dehydrogenase (G6PD)/HIF-1α signaling, consequently conferring the proliferation and metastasis of breast cancer cells via EMT signaling (epithelial–mesenchymal transition) due to extensive NADPH levels [144]. Furthermore, Nrf2 could also promote the PPP gene (Serine/threonine phosphatase, PPP) expression in prostate and lung cancer cells due to a Keap1 loss-of-function mutation that fosters tumor growth and metastasis through the upregulation of G6PD, transketolase (TKT), and phosphogluconate dehydrogenase (PGD) and the epigenetic impairment of miR-1 and miR-206 [145]. Therefore, the therapeutic modalities targeting Nrf2 signaling should be developed to block the activity of Nrf2 in cancer prevention.

### 4.3. Glycation induced AGE-RAGE Signaling and Nrf2

Nrf2-Keap1 complex formation significantly promotes the uncontrolled proliferation of cancer cells, followed by the metastasis, via the regulation of p53-mediated apoptotic signaling [21,146]. AGEs can regulate Nrf2 and ERK phosphorylation to control downstream pathways. For instance, AGEs could potentially inhibit Nrf2, p53, and Bcl2-x (an apoptosis regulator) and control oral cancer cell survival through ERK phosphorylation [21]. In addition, multidrug resistance to the chemotherapeutic agents in tumor cells was promoted through Nrf2 signaling during high glycolytic activity and Glo1 expression [45]. Extensive research studies are still required to study the modulatory effect of AGE–RAGE signaling in the Nrf2 pathway to develop novel therapeutic targets that can mitigate tumor progression.

### 4.4. Global Inhibition of Deglycation and Nrf2: Limitations

The mutations pertaining to Nrf2 activation exhibit significant impact on the efficacy of chemotherapy, which further suggests the urgent need for the development of specific therapeutic modalities to target Nrf2. Targeting protein glycation is one approach, which requires additional research. Protein glycation is a nonenzymatic post-translational reaction of reducing sugars like ribose, fructose, and glucose-6-phosphate with basic amino acids, viz., lysine, arginine, and the histidine of proteins like Nrf2 to generate fructosamines in a Maillard reaction [117,147,148]. These basic amino acids residing in the accessible and functionally relevant domains are often being affected by glycation; therefore, any glycation-induced alterations in structure and charge may affect protein functions involved in cancers (for example, Nrf2) [117,149].

Deglycation is the removal of sugars from the proteins by FN3K, a well-known kinase reported to be involved directly in phosphorylating the attached sugar and destabilizes the protein [149]. In normal conditions, Nrf2 is active in cancer cells to protect them from redox stress by triggering the activation of AREs to produce antioxidant responses during chemotherapy or inner immune cues [87]. The catalytic activity of the FN3K deglycating enzyme has a significant role in cancer cells to modulate the oncogenic activity of Nrf2, as the blockade of FN3K could induce glycated Nrf2 to remain Nrf2 in an inactive state (Figure 3B) [87]. However, glycation and enzymatic glycosylation are different, since enzymatic glycosylation facilitates a much slower formation of AGEs implicated in metabolic diseases like diabetes and inflammatory conditions [117,150,151,152]. For example, the formation of glycated protein can be seen in the hemoglobin HbA1c of erythrocytes, which can track the glucose level in the human blood [153]. Certain other examples of glycation-derived products are glycated insulin and serum albumin [154,155,156]. Still, future research should uncover the effects of glycation on Nrf2-mediated signaling cascades and on other cellular proteins to develop NSMIs against cancers.

A recent report by Sanghvi et al. (2019) vividly delineated the mechanism of Nrf2 activation in hepatocellular carcinoma (HCC) development and progression [87]. The study reported that the activity of Nrf2 relies upon FN3K, a kinase enzyme needed for Nrf2 deglycation [87]. This study showed the knockdown of FN3K led to glycated Nrf2, which further mitigated HCC development due to the lack of binding sites to bind to sMAF proteins. However, the global inhibition of deglycation has yet to be extensively reported in all other cancer types with selectivity and tissue specificity to develop novel FN3K inhibitors to maintain Nrf2 in a glycated state or to modulate the role of Nrf2 activity in cancers. Several studies already evaluated and validated the specificity and selectivity of Nrf2 modulators against several cancers. For instance, a report by Anju Singh et al. (2016) evaluated and validated the selective and specific delivery of ML385, a specific Nrf2 inhibitor in combination with carboplatin, in NSCLC-A549 cells with Keap1 mutation, and this nanoformulation delivered effective preclinical results as a promising strategy to treat Nrf2-mediated NSCLC [157]. In this context, the development novel FN3K inhibitors in combination with current chemotherapeutic molecules as nanoformulations may deliver selectivity and specificity in promoting the formation of glycated Nrf2 for treating several Nrf2-mediated cancers.

## 5. Fructosamine Kinases (FN3K and FN3K-RP) in the Regulation of Cancers

FN3K is abundant in both eukaryotes and prokaryotes, since they contain a single copy of the FN3K gene [158]. However, mammalian cells are composed of two copies of genes that code for FN3K and the FN3K-related protein (FN3K-RP) [149,158,159,160]. FN3K is highly conserved across various living beings to maintain cellular homeostasis by mitigating AGE-mediated oxidative stress [161]. Prospective research should focus on uncovering the fundamental role and regulation of FN3K in several cellular functions and disease models [162]. FN3K is more abundantly found in the liver, heart, kidney, brain, and skeletal muscles and relatively higher in erythrocytes [158]. FN3K is composed of 309 amino acids and coded from chromosome17q25.3 of the human genome [86,163]. Isoforms of FN3K and the fructosamine-3-kinase-related protein (FN3KRP) are observed in eukaryotes, including mammals, birds, amphibians, fishes, and nematodes. However, FN3K expression was not found in yeasts, arthropods, and Drosophila [163]. RNA interference knockout studies in *Caenorhabditis elegans* for FN3K activity were proven beneficial in determining its function at the cellular level [164,165].

Glycation is a significant nonenzymatic process occurring in the majority of living cells, but deglycation is an enzyme-driven reaction [161]. One such deglycation mechanism involves the activity of fructosamine kinases, viz., FN3K and fructosamine-6-kinase (FN6K); both enzymes are distinct in catalytic mechanisms [161]. FN3K in humans could effectively cleave fructosamines in a two-step process, which starts with C3 phosphorylation at a fructosyl moiety, followed by the production of unstable fructosamine 3-phosphate. Next, this molecule generates 3-deoxyglucosone through autocatalytic degradation [161]. FN3K and FN3K-related homologs are reported as sensing molecules in several organisms [161]. FN3K-related proteins (FN3K-RP) were significantly involved in reacting with C3 epimers, viz., psicosamines, ribulosamines, and erythrulosamines [161,166,167].

FN3K is an ATP-dependent protein repair enzyme mainly found in Aves and mammals, whereas fructoselysine-6-phosphate deglycase (FL6PDG) is reported in bacteria [85,168,169]. FN3K-RP could catalyze the phosphorylation of Amodari products, viz., ribulosamines and psicosamines, whereas FN3K catalyzes fructosamine formation [158]. Blast sequence searches in chordate genomes identified two genes encoding for proteins homologous to FN3K or FN3K-RP in several mammals. Only one gene was reported to encode a protein homologous to FN3K-RP rather than FN3K in fish and *Ciona intestinalis* [158]. FN3K is involved in mediating protective roles against ribose-induced apoptosis of pancreatic islets, suggesting that this enzyme activity is significantly attributed to offer protection against oxidative stress [170].

FN3K and FN3K-RP exhibit nearly 65% sequence similarity [86,171]. Knockout studies to suppress FN3K expression in mice models have reported the accumulation of protein-bound fructosamines, suggesting the physiological role of FN3K in modulating early glycation adducts in vivo [151]. FN3K-RP failed to phosphorylate protein-bound fructosamines; however, it can induce the glycation of ribulosamines or psicosamines [167]. In human erythrocytes, FN3K mediates the phosphorylation of sorbitol or fructose and generates sorbitol-3-phosphate and fructose-3-phosphate, respectively [86,172,173]. Thus, both FN3K and FN3K-RP possess unique substrate specificity to phosphorylate protein-bound ketosamines [174]. BLAST searches demonstrated a set of proteins involved in encoding microbial sequences that share nearly 30% sequence homology with human FN3K [85]. For example, the aminoglycoside kinases in microbial species could confer bacterial antibiotic resistance, and this enzymatic protein is reported to possess 30% sequence homology with human FN3K [175,176,177]. Sequence alignment studies represented that these ketosamine kinases exhibit many conserved residues, viz., Lys41, Glu55, and Asp244 and a conserved DxxxxN motif from 227 to 232 in the FN3K-RP primary structure [176].

FN3K actively phosphorylates CS-0777, an active S1P1 agonist reported to act against multiple sclerosis [178]. CS-0777 is a sphingosine 1-phosphate receptor modulator that produces M1 metabolites upon FN3K activity [178]. The first deglycating mechanism concluded the efficacy of fungal amadoriases in the hydrolysis of fructosamines [17]. It is crucial to unravel whether deglycation is a significant antioxidant defense mechanism/epiphenomenon by examining its enzymatic activity towards substrates in several cancers [81]. The physiological occurrence of deglycation prominently occurs in tissues with a high concentration of protein-bound fructosamines [179,180]. Thus, it is plausible to recycle FN3K activity in the deglycation pathway to mitigate AGE formation during metabolic diseases [81].

FN3K mediates the phosphorylation of fructoselysine residues in glycated proteins, resulting in the generation of protein-bound fructolysine-3-phosphate in human tissue [180]. A report by Misciagna G et al. (2004) delineated the role of carbonyl stress to induce the malignant cell transformation of colorectal adenomas, where serum fructosamine levels play a significant role in promoting malignant transformation [180,181]. Hence, deglycation pathways mediated by FN3K have plausible significance in certain cancers, such as colorectal carcinomas [181]. The differential expression of FN3K in cancer cells rather than normal cells has significant implications in the pathophysiology of several cancers [182]. For instance, a report by M. Notarnicola et al. (2010) evaluated the expression of FN3K in colorectal cancer patients and concluded that the expression of FN3K gene is comparatively lesser in colon cancer tissue than the adjacent normal mucosa [180]. This report concluded that FN3K expression is particularly required for the deglycating enzyme system in colon cancer [180]. On the contrary, a report by Shangvi et al. (2019) concluded that the activity of FN3K should be blocked in the case of HCC. Functionally active FN3K makes oncogenic Nrf2 inactive by retaining it in a glycated state, suggesting the intricate and versatile role of FN3K in cancers [87]. These glycated proteins exhibit a reduced half-life and are often devoid of substrate activity, consequently recognized as misfolded and directed to proteasomal degradation. Hence, novel therapeutic agents to modulate the enzymatic activity of FN3K are imperative for individual cancers by determining the specific role of FN3K for every cancer. However, the application of genomics/transcriptomics/proteomics-centric approaches as multi-OMICS strategies may deliver key insights into the complex role of FN3K in several individual cancers to develop gene-based therapies to modulate the expression of FN3K.

The functional aspects of FN3K exclusively rely on its conserved structural motifs in this protein. For instance, the redox-sensitive P-loop Cys is highly conserved amongst FN3K orthologs in both prokaryotes and eukaryotes [162]. The efficient catalysis of FN3K in delgycating the glycated proteins depends on the P-loop, which mainly consists of a GlyxGlyxxGly motif. This motif is primarily conserved in diverse ATP enzymes to foster conformational flexibility during catalysis [162]. A report by Safal Shrestha et al. (2020) delineated that FN3K is composed of Gly residues, as well as Cys residues, in the P-loop. The authors of this study reported that tyrosine protein kinases were also composed of conserved Cys residues similar to FN3K in the Gly-rich motifs of P-loop [162]. For example, the presence of Cys at the position Cys32 of FN3K can be observed in the tyrosine kinases of eukaryotes, viz., SRC, FGFR (human fibroblast growth factor receptor), YES1 (YES proto-oncogene 1), and FYN tyrosine kinases [162]. The expression of both FN3K and FN3K-RP with Cys-rich motifs is highly abundant in human tumors [162]. However, the development of therapeutic modalities for FN3Ks is significantly a double-edged sword, because “the blockade of FN3K might cause the accumulation of glycated proteins, whereas the activation of FN3K might cause the accumulation of 3-deoxyglucosone”. The latter one generates extensive oxidative stress [162].

The mutation studies of Cys32Ala/Cys236Ala/Cys196Ala of FN3K revealed the existence of both dimeric and monomeric species, suggesting that this enzyme can potentially undergo dimerization without these cysteines [162]. Thiol-oxidizing agents like diamide altered the dimerization and higher-order olgomerization of FN3K [162]. Another study by S. Akter et al. (2018) reported sulfenylation at the P-loop Cys of human FN3K-RP in HeLa cells during oxidative stress [162,183]. The results of this study suggest that partial Cys P-loop oxidation to sulfenic acid is a reversible modification, which could be a regulatory mechanism for FN3K operating in cells [183]. Further, redox-active Cys in FN3K orchestrates the possibility of a feedback regulatory mechanism for FN3K, as its activity can be controlled by 3-deoxyglucosone (3-DG), a catalytic byproduct of FN3K. Prior studies have shown the potential of 3-DG to contribute to oxidative stress in cells [184]. The accumulation of AGEs fosters the conformational assembly of FN3K towards an inactive dimeric form by P-loop Cys oxidation, while the decline in AGEs would result in the FN3K in an active-reduced form [162]. This kind of feedback inhibition is a regulatory mechanism of FN3K essential for the delgycation of proteins inside cancer cells/normal cells during oxidative stress. In this scenario, it is imperative to uncover the regulatory mechanism for the redox-active switch/feedback regulation of FN3K in deglycating Nrf2 in cancer cells. The comparative metabolomics/biochemical and mutational studies pertaining to FN3K activity would benefit combinatorial drug development strategies to target both active monomeric/reduced dimeric species of FN3K by developing thiol-interacting agents [162].

## 6. Cross-Talk between Nrf2 and FN3K-Mediated Deglycation

FN3K is a proven drug target to mitigate Nrf2-driven cancers [87]. Accumulating evidence has shown that Nrf2 is activated by mutations in 30% of tumors, viz., lung, liver, head, neck, oral, pancreatic, etc. This transcription factor has a specific oncogenic role in these cancers by modulating redox signaling [105,109,110,119,185].

FN3K is actively involved in promoting cell signaling to mitigate oxidative stress and foster cellular repair, exhibiting defense mechanisms against toxins with distinct transcriptional regulation [85,86,90,186,187]. Oncogenic Nrf2 function requires the removal of sugars, a process induced via deglycation reactions catalyzed by FN3K [87]. Sanghvi et al. (2019) delineated the sensitivity of oncogenic Nrf2 to glycation. The authors of this novel study reported a pivotal role for FN3K in regulating Nrf2 activity during cancer progression [87]. FN3K is the unique kinase that eliminates sugars from binding to proteins (ex., Nrf2) spontaneously [149,188]. Glycation could foster Keap1-mediated Nrf2 degradation. Moreover, it was concluded that Nrf2 glycation blocks its interaction with other transcription co-factors, viz., sMAF proteins. This was studied in both Keap1-proficient and mutant cells [87]. Cellular metabolites can directly intervene and influence the Nrf2 activity in cancer cells [189,190]. For instance, a mass spectrometry analysis confirmed the presence of sugar adducts on Nrf2 in HCC cells [87]. The C-terminus of Nrf2 exhibited 50% glycation in the absence of FN3K [87]. Moreover, glycation can occur autonomously across all the sites; however, the fraction of glycated Nrf2 may be higher in cellular environments. The glycation kinetics of Nrf2 is significantly in line with the glucose concentration and Nrf2 half-life in cancer cells [148,191]. Cellular metabolites could also influence this deglycation pathway; for instance, methylglyoxal and itaconate are the byproducts generated from glycolysis and the Krebs cycle, respectively, which can intervene and influence Keap1 to consequently modify Nrf2 function [189,190].

The generation of glycated proteomes inside cancer cells solely depends on the glycatibility of a spatial or chemical nature of basic amino acids of proteins like Nrf2 and their sensitivity to FN3K-mediated deglycation [192,193]. Earlier studies by Nokin et al. (2016) reported that the metabolic enzymes, viz., lactate dehydrogenase A (LDHA), lactate dehydrogenase C (LDHC), eukaryotic initiation factor 4A-I (elF4A1), eukaryotic translation initiation factor 1 (eIF1), eukaryotic translation initiation factor 3 subunit G (eIF3G), and heat shock protein (HSP)-90, undergo glycation. On a similar note, Sanghvi et al. (2019) reported that nearly 100 proteins underwent FN3K-sensitive glycation [194]. For instance, HSP90AA1 and HSP90AA4; DNA and RNA-binding proteins like Nrf2; DNA replication and repair protein, viz., DNA helicase B (HELB), minichromosome maintenance complex component 3 (MCM3), splicing factors like serine/arginine-rich splicing factor 7 (SRSF7), PUF60 (Poly(U)-binding splicing factor 60), and H3 and H4 histone proteins underwent extensive glycation in the cellular environment under different physiological conditions [195,196]. Prospective research should focus on the mechanisms underlying the other cellular modifications of histone glycation sites, viz., H3K115 and H2BK108 [196]. For example, tumor cells are characterized by a high glucose uptake to generate lactate in the mitochondria, where a glucose-derived carbonyl reactive species MGO (methyl glyoxal) is excessively generated [197]. MGO is reported to be a strong glycating agent that can modify proteins and DNA, which culminates into the progression of cancers. MGO is also reported to be involved in histone and DNA modification, which, consequently, alters gene expression and DNA repair [196,197,198,199]. The Nrf2 protein is susceptible to post-translational sugar modification, indicating that glycation significantly affects the functional aspects of other cellular proteins [87].

Thus, potential drug development strategies should focus on targeting FN3K-mediated Nrf2 redox signaling and their downstream signaling cascades that are activated in 30% of tumors, viz., lung, liver, head, neck, oral, pancreatic, etc. [105,109,119,185]. The occurrence and progression of HCC in vivo models is driven by Myc and Keap1 inactivation, which further relies upon the activity of FN3K [87]. According to the TCGA data, the functions of less frequent somatic mutations in Nrf2 glycation sites, viz., R499W, R569C, and R569H are yet to be investigated in cancers such as colorectal cancer, endometrial cancer, and melanomas. NSMIs targeting vulnerable FN3K could be an effective strategy to maintain Nrf2 in an inactive state; this was concluded from a genomic study in FN3K knockout mice models, where the data supported a specific need of FN3K for oncogenic Nrf2-driven lung and liver carcinomas [87,151].

## 7. Need for the Development of FN3K Inhibitors against Breast Cancer

Breast carcinomas are the leading cause of morbidity and mortality in women across the world [200,201,202,203]. Nearly eight million deaths were reported in the year 2008 due to breast cancer, which is expected to rise to 11 million by 2030 [204]. In the year 2018, about two million new breast cases were reported accounting for ~23% of all cancers. The incidence rate of breast cancer is nearly 19.3 per 100,000 women in Eastern Africa, whereas the incidence rate in Western Europe is 89.7 per 100,000 women [205]. The reasons for the substantial rise in breast cancer cases worldwide are industrialization, ageing, severe population growth, and carcinogenic pollutants [206]. On the other hand, alcohol consumption, cigarette smoking, lifestyle and changing dietary patterns, childhood obesity, and socioeconomic status further contribute to the increasing incidence of breast cancer (BC) cases [206]. Recent epidemiological studies reported a 11.54% increase in incidence and a 13.8% increase in the mortality rate in breast cancer patients, necessitating the development of immediate intervention strategies [206].

The accumulation of genetic/epigenetic alterations in human breast epithelial cells triggers the transformation of normal cells into breast cancer cells [206,207]. Transformed breast cancer cells become aggressive and begin to invade other organs [207]. Invasive breast carcinomas are the major contributors for a higher mortality rate, as the existing therapies are either minimally effective or a rapid drug resistance was developed for various existing chemotherapies [208]. Therefore, identifying a protein that regulates not only the proliferation and metastasis of cancer cells but also, the drug resistance is a significant finding. Recently, studies from our laboratory identified the upregulation of Nrf2 in malignant breast carcinomas and demonstrated that the targeted inhibition of Nrf2-sensitized cancer cells to chemotherapeutic agents (Bovilla et al., unpublished results). The activation of Nrf2 is associated with the overexpression of MRP1-5 and breast cancer resistance protein (BCRP) genes. Hence, the growing interest towards the development of therapeutic modalities to target the oncogenic functions of Nrf2 are under intense investigation [87,209].

The role of Nrf2 in breast cancer: Studies from several laboratories have reported the involvement of Nrf2 in breast cancer development, drug resistance, and malignant behavior [210]. For instance, a recent study reported that Nrf2 modulates RhoA expression and causes the progression of BC. Another report by Kevin Lu et al. (2017) demonstrated the role of dipeptidylpeptidase-3 (DPP3) overexpression in modulating Nrf2/Keap1 signaling, which led to a chemotherapeutic drug resistance and progression of BC [211]. A genomics study described the active role of oncoprotein HBXIP (mammalian hepatitis B X-interacting protein) in regulating Nrf2/Keap1 activity and the growth and metastasis of BC [212]. Recently, the role of Nrf2/keap1 in modulating the expression of Notch1 via G6PD/HIF-1α and the EMT pathway was described in the proliferation of BC cells [213].

However, to date, no studies have reported the role of glycation in the regulation of Nrf2 in breast cancer cells. FN3K is a unique protein that mediates deglycation by phosphorylating the basic amino acids lysine, arginine, etc. in Nrf2. The FN3K-sensitive glycation of several hepatic proteins, such as translation factors, DNA replication and repair proteins, splicing factors, and histone proteins, has been reported in several cancers [91]. Yet, it is imperative to examine the expression of FN3K in human breast cancer cells. The activation of Nrf2 leads to the development of many solid tumors in various organs that include the liver, lung, brain, and pancreas [102,103,209,214]. Hence, therapeutic modalities such as NSMIs or phytochemicals in combination therapy to target the FN3K-mediated modulation of Nrf2 glycation should be developed to maintain Nrf2 in an inactivated state [87].

The glycation kinetics of Nrf2, including the half-life of Nrf2, is significantly related to the levels of monosaccharides in cancer cells. Studies have demonstrated the key role of FN3K in the glycation of proteins and accumulation of AGEs in several cancers [87]. However, Nrf2 protein deglycation in cancers is mediated by FN3K, concluding that the activity of Nrf2 needs FN3K. Another report described the role of methylglyoxal generated from glycolysis and itaconate from citric acid cycle on Keap1-Nrf2 signaling [189]. Therefore, the targeted inhibition of FN3K using small molecule inhibitors is a viable strategy to inhibit breast cancer.

## 8. Phytochemical and Drug Interventions to Modulate Glycation of Proteins

The inhibition of protein glycation using antioxidant phytochemicals: Phytochemical antioxidants such as vitamin C and E, lycopene, and carotenoids are known to mitigate protein glycation in various in vitro and in vivo models [215]. Leaf extracts from *Ziziphus oxyphylla* and *Cedrelas errata* confer anti-glycating effects, and their formulation in a mixture exerted an anti-glycating effect rather than individual extracts and quercetin (a reported anti-glycating agent) [216]. Another report by Ahmad et al. (2016) delineated the anti-glycating effect of unripe fruits from *Coccinia grandis* [5,217]. A study by Raghu et al. (2017) reported that ellagic acid alleviated retinal complications, viz., angiogenesis, hypoxia, and cell damage, by blocking the generation of AGE–RAGE-mediated cellular cascades [218]. Other phytoconstituents from North American cranberry fruits exhibited anti-glycating effects by blocking AGE production [219]. Similarly, extracts from Maple syrup also blocked the production of AGEs and neutralized free radicals, consequently inducing an anti-glycating effect to protect normal/nontumorigenic human colon CCD-18Co cells by activating cell survival proteins, viz., pERK [220]. The bark methanol extract isolated from *Eysenhardtia polystachya* could confer multitarget effects, such as anti-glycation, anti-hyperglycemia, and hepatoprotectivity in diabetic mice [221]. Cinnamic acid and its derivatives were reported to exhibit fructose-mediated protein glycation and protect BSA (bovine serum albumin) in vitro; these derivatives were proven effective for the management of protein glycation, diabetic complications, and AGE-mediated pathologies [222]. In addition, cinnamic acid significantly mitigated the fructosamine levels and protein carbonyl contents, suggesting its efficacy in reducing carbonyl and glycativ stress [222]. The activity of these molecules also should be examined against the FN3K-mediated deglycation of Nrf2 in several cancers. Several herbal extracts from ginger, cinnamon, cloves, and tarragon that are composed of polyphenols, viz., luteolin, quercetin, and rutin, could block the glycation of BSA in vitro models [223]. The efficacy of these polyphenols must be determined in mediating FN3K-mediated deglycation for the development of NSMIs against breast cancer.

Papavarine is an opium alkaloid isolated from *Papaver somniferum* and reported to be effective for mitigating fibroblastomas by blocking the activity of HMGB1 and blocking its downstream RAGE-mediated NF-kB signaling [20]. Furthermore, this alkaloid can also block the proliferation of glioblastoma cells by downregulating HMGB1 and RAGE [224]. Low molecular weight heparin has been shown to block HMGB1-induced NF-kB activation through RAGE [225]. Chondroitin sulfate also has been proven effective to block the activity of RAGE and mitigated pulmonary metastasis [226]. Hispidin is a natural polyphenol isolated from *Phellinus linteus* against colon cancer, and its combination with ergothioneine blocked the activation of RAGE expression and NF-kB activation [227]. Ethyl pyruvate is another effective HMGB1 blocker and is proven effective against malignant mesothelioma in vitro and in vivo models [228] by inducing the blockade of RAGE and NF-kB expression. Further, it could also block the proliferation of NSCC cell growth, invasion, and migration by downregulating the HMGB1-RAGE pathway and NF-kB-STAT3 pathway [229] (Table 2).

A recent patent by Benjamin Szwergold, 2012, described the role of α-thiolamine in inhibiting the enzymatic glycation of proteins (patent number: 8138227); however, thiolamine was found to be ineffective pharmacologically (patents by Inventor Benjamin Szwergold: Method for inhibiting or reversing non-enzymatic glycation, patent number: 8138227). The high-throughput screening (HTS) of several novel conventional small molecule inhibitors and phytochemicals targeting FN3K is highly significant and contributes to the development of novel inhibitors for targeting oncogenic Nrf2-driven cancers. In this context, future research should focus on the development of therapeutic modalities targeting FN3K in Nrf2-driven cancers using molecular docking models in in vitro, in vivo, and clinical studies.

## 9. Conclusions

The deglycation of Nrf2 is catalyzed by FN3K, whose activity is enhanced during the progression of cancer cells into malignant and drug-resistant phenotypes. Thus, the deglycation of Nrf2 is one of the significant mechanisms that might be contributing, in part, to the tumorigenic potential of cancer cells. However, the current research reporting on the deglycation of Nrf2 requires extensive research not only to determine the role of FN3K in cancers but, also, to develop novel pharmacological agents to modulate the expression of glycated Nrf2 in cancers. Hence, future studies should develop strategies to target FN3K either alone or in combination with Nrf2 for effective tumor retardation. As AGEs are reported to be involved in both diabetes and cancer, it is crucial to ascertain the pharmacological efficacy of existing therapies against AGE-mediated cancers.

## Figures and Tables

**Table 1:** Various signaling pathways affected by receptors for advanced glycation end products (RAGEs) in cancer progression.

Signaling Pathways Affected by RAGEs	Regulation	Cancers	Refs.
p53	Downregulation	Cancer cell proliferation (oral cancer, prostate cancer, pancreatic carcinoma, and erythroleukemia)	[21]
Beclin 1	Upregulation		[22]
JAK/STAT3	Upregulation		[23]
MAPKs	Upregulation		[23]
PI3K/Akt/mTOR	Upregulation		[24]
NF-kB	Upregulation		[25]
VEGF	Upregulation	Angiogenesis (Breast cancer)	[25]
MMPs	Upregulation	Metastasis (Human breast and gastric tumors)	[13,26]
Nrf2	Downregulation	Human oral cancer	[21]

JAK: Janus kinase, MAPKs: mitogen-activated protein kinases, PI3K: phosphoinositide-3-dependent kinase, mTOR: mammalian target of rapamycin, NF-kB: nuclear factor of kappa beta, VEGF: vascular endothelial growth factor, MMPs: matrix metalloproteinases/matrix metallopeptidases, and Nrf2: nuclear factor erythroid-2 related factor 2.

**Table 2:** Phytochemicals and drug interventions that modulate AGE-RAGE-mediated glycation to mitigate cancer cell growth.

Phytochemical	Signaling Pathways	Cancers	Refs
Papavarine	Downregulation of HMBG1, RAGE, and NF-κB	Fibrosarcoma	[20]
	Downregulation of HMBG1 and RAGE	Glioblastoma	[224]
Cinnamic acid	-	Yet to be examined against AGE-RAGE-mediated cancers (?)	[222]
Ellagic acid	-		[218]
Maple syrup	-		[220]
Thiol-amine	-		
**Drugs**			
Ergothioneine	Downregulation of AGEs, RAGE, and NF-κB	Pheochromocytoma	[227]
Hispidin		Pheochromocytoma	[227]
Chondroitin sulfate and heparan sulfate	-	Lung metastasis	[226]
Duloxetine	Downregulation of S100B	Glioma cancer	[230]
Ethyl pyruvate	Downregulation of HMBG1, RAGE, and NF-κB	Malignant mesothelioma	[228]
	Downregulation of the HMBG1, RAGE, NF-κB, and STAT3 Pathways	Non-small cell lung cancer	[229]
Low molecular weight heparin	Downregulation of RAGE-mediated NF-kB	Fibrosarcoma	[225]

## Abbreviations

3-DG	3-deoxyglucosone
4-HNE	4-hydroxynonenal
AGEs	Advanced glycation end products
AP-1	Activator protein 1
ARE	Antioxidant response element
BC	Breast cancer
BCRP	Breast cancer resistance protein
BSA	Bovine serum albumin
CAND1	Cullin-associated NEDD8-dissociated protein 1
ChREBP	Carbohydrate response element binding protein
CUL3	Cullin3
eIF1	Eukaryotic translation initiation factor 1
eIF3G	Eukaryotic translation initiation factor 3 subunit G
elF4A1	Eukaryotic initiation factor 4A-I
eNOS	Endothelial nitric oxide synthase
EMT	Epithelial–mesenchymal transition
FGFR	Human fibroblast growth factor receptor
FL6PDG	Fructoselysine-6-phosphate deglycase
FN3K	Fructosamine-3-kinase
FN3K-RP	Fructosamine-3-kinase-related-protein
FN6K	Fructosamine-6-kinase
GCLC	Glutamate-cysteine ligase catalytic subunit
GCLM	Glutamate cysteine ligase modifier subunit
GPx	Glutathione peroxidase
GSH	Glutathione
GST	Glutathione S-transferase
HbA1c	Glycated hemoglobin
HBXIP	Mammalian hepatitis B X-interacting protein
HCC	Hepatocellular carcinoma
HELB	DNA helicase B
HIF-1α	Hypoxia-inducible factor 1-alpha
HMGB1	High mobility group box-1
HO1	Heme oxygenase-1
HSP	Heat shock protein
HSP90AA1	Heat shock protein 90 alpha family class A member 1
HSP90AA4	Heat shock protein 90 alpha family class A member 4
ICAM-1	Intercellular Adhesion Molecule 1
IL-1β	Interleukin-1β
IL-6	Interleukin 6
JAK	Janus kinase
Keap1	Kelch-like ECH-associated protein 1
KRAS	Kirsten rat sarcoma viral oncogene homologue
LDHA	Lactate dehydrogenase A
LDHC	Lactate dehydrogenase C
MAF	Musculoaponeurotic fibrosarcoma
Maf	Musculoaponeurotic fibrosarcoma
MAPK	Mitogen activated protein kinase
MCM3	Minichromosome maintenance complex component 3
MD	Malondialdehyde
MGO	Methylglyoxal
MMPs	Matrix metalloproteinases/Matrix metallopeptidases
mTOR	Mammalian target of rapamycin
NAD(P)H	Reduced nicotinamide adenine dinucleotide phosphate
NFE2L2	Nuclear factor erythroid-derived 2-like 2 gene
NF-κB	Nuclear factor kappa—B
NOX	NADPH oxidases
NQO1	NAD(P)H:quinone oxidoreductase 1
Nrf2	Nuclear factor erythroid 2 (NFE2)-related factor 2
NSCLC	Non small cell lung carcinoma
NSMIs	Novel small molecule inhibitors
p53	Tumor protein p53/phosphoprotein p53/tumor suppressor p53
pERK	Protein kinase R-like endoplasmic reticulum kinase
PI3K	Phosphoinositide 3-kinase
PNG-1	Peptide N-Glycanase 1
PPAR-γ	Peroxisome proliferator-activated receptor gamma
PUF60	Poly(U)-binding-splicing factor
RAGEs	Receptors for advanced glycation end products
RNS	Reactive nitrogen species
ROS	Reactive oxygen species
SCLCs	Squamous cell lung carcinomas
sMaf	small Maf proteins
SOD	Superoxide dismutase
SRSF7	Serine/arginine-rich splicing factor 7
STAT3	Signal transducer and activator of transcription 3
TAMs	Tumor-associated macrophages
TCGA	The cancer genome atlas
TNF	Tumor necrosis factor
TNFR-1	Tumor necrosis factor receptor 1
TNF-α	Tumor necrosis factor alpha
Txn	Thioredoxin
VCAM-1	Vascular cell adhesion molecule 1
VEGF	Vascular endothelial growth factor
YES1	YES proto-oncogene 1
